# Prospective multicentre cohort study of heparin-induced thrombocytopenia in acute ischaemic stroke patients

**DOI:** 10.1111/j.1365-2141.2011.08775.x

**Published:** 2011-06-14

**Authors:** Hiroyuki Kawano, Haruko Yamamoto, Shigeki Miyata, Manabu Izumi, Teruyuki Hirano, Naomi Toratani, Isami Kakutani, Jo-Ann I Sheppard, Theodore E Warkentin, Akiko Kada, Shoichiro Sato, Sadahisa Okamoto, Kazuyuki Nagatsuka, Hiroaki Naritomi, Kazunori Toyoda, Makoto Uchino, Kazuo Minematsu

**Affiliations:** 1 Department of Cerebrovascular Medicine, National Cerebral and Cardiovascular CentreSuita, Osaka; 2 Department of Neurology, Faculty of Life Sciences, Kumamoto UniversityKumamoto; 3 Department of Advanced Medical Technology Development, National Cerebral and Cardiovascular CentreSuita, Osaka; 4 Divisions of Clinical Laboratory and Transfusion Medicine, National Cerebral and Cardiovascular CentreSuita, Osaka; 5 Division of Cardiology, Department of Internal Medicine, Research Institute for Brain and Blood Vessels AkitaAkita; 6 Department of Neurology, National Cerebral and Cardiovascular CentreSuita, Osaka, Japan; 7 Department of Pathology and Molecular Medicine, McMaster UniversityHamilton, ON, Canada

**Keywords:** acute stroke care, anticoagulation, heparin, platelet, thrombocytopenia

## Abstract

Acute ischaemic stroke patients sometimes receive heparin for treatment and/or prophylaxis of thromboembolic complications. This study was designed to elucidate the incidence and clinical features of heparin-induced thrombocytopenia (HIT) in acute stroke patients treated with heparin. We conducted a prospective multicentre cohort study of 267 patients who were admitted to three stroke centres within 7 d after stroke onset. We examined clinical data until discharge and collected blood samples on days 1 and 14 of hospitalization to test anti-platelet factor 4/heparin antibodies (anti-PF4/H Abs) using an enzyme-linked immunosorbent assay (ELISA); platelet-activating antibodies were identified by serotonin-release assay (SRA). Patients with a 4Ts score ≥4 points, positive-ELISA, and positive-SRA were diagnosed as definite HIT. Heparin was administered to 172 patients (64·4%: heparin group). Anti-PF4/H Abs were detected by ELISA in 22 cases (12·8%) in the heparin group. Seven patients had 4Ts ≥ 4 points. Among them, three patients (1·7% overall) were also positive by both ELISA and SRA. National Institutes of Health Stroke Scale score on admission was high (range, 16–23) and in-hospital mortality was very high (66·7%) in definite HIT patients. In this study, the incidence of definite HIT in acute ischaemic stroke patients treated with heparin was 1·7% (95% confidence interval: 0·4–5·0). The clinical severity and outcome of definite HIT were unfavourable.

Immune-mediated heparin-induced thrombocytopenia (HIT), which is caused by platelet-activating IgG antibodies that recognize platelet factor 4 bound to heparin (anti-PF4/heparin Abs), is a relatively common side effect of heparin therapy and presents a strong risk factor for thromboembolic events associated with high mortality and morbidity (Warkentin, 2007a). Prospective studies in Western countries have shown that the prevalence of HIT is 0·3–5% of patients treated with unfractionated heparin (UFH), which varies depending on the clinical settings (Warkentin *et al*, 1995, 2000; Kappers-Klunne *et al*, 1997). Thrombotic complications occur in approximately one-third to one-half of HIT patients (Warkentin, 2007a). On the other hand, some studies of UFH therapy for acute stroke reported no cases of HIT (Toth & Voll, 2002; Camerlingo *et al*, 2005). To elucidate the prevalence of HIT in acute ischaemic stroke patients who were treated with heparin, we organized a prospective multicentre cohort study that included systematic collection of blood for detection of the antibodies that cause HIT.

Some clinical guidelines do not recommend prescribing heparin in acute ischaemic stroke, and others recommend it mainly for the prevention of deep vein thrombosis (DVT) and pulmonary embolism (PE) (Albers *et al*, 2004; Cardiovascular Disease Educational and Research Trust, 2006; Adams *et al*, 2007). At the participating stroke centres in our study, in addition to the prevention of DVT and PE, UFH is given during the acute phase of ischaemic stroke to the following: patients with emboligenic heart disease or superimposed thrombi on the carotid plaque to prevent embolic complications; patients with particular stroke aetiologies, including cerebral arterial dissection and vasculitis; and patients with embolic stroke of unknown origin until the presence of heart disease is excluded by the results of prolonged electrocardiography and transesophageal echocardiography (Caplan, 2003).

In a previous study of 137 stroke patients who were treated with UFH, 21 patients (15·3%) developed thrombocytopenia (≥40% fall in platelet counts) during or after heparin therapy, and five of these 21 patients had an additional ischaemic stroke (Ramirez-Lassepas *et al*, 1984). A recent study of 200 neurological patients treated with UFH for at least 5 d, including 102 patients with cerebrovascular disorders, demonstrated that 41 patients (20·5%) had anti-PF4/heparin Abs and 5 (2·5%) developed HIT, when the serological diagnosis was made from the presence of antibodies detected by an enzyme-linked immunosorbent assay (ELISA) (Harbrecht *et al*, 2004).

Only a few studies have investigated the prevalence of HIT in acute stroke patients receiving UFH, especially in the Asian population (Kawano *et al*, 2008). In our previous retrospective report of acute ischaemic stroke patients who were treated with UFH, 0·5% of the patients developed HIT diagnosed by both the clinical scoring systems and the serological assays, including ^14^C-serotonin release assay (SRA) (Kawano *et al*, 2008). However, our retrospective study assessing the prevalence of HIT was limited by the fact that was that antibodies were not assayed in all patients. This limitation may cause an under diagnosis of HIT.

Thus, we performed this prospective multicentre cohort study in 267 patients to determine a more accurate incidence of HIT in patients with acute ischaemic stroke and to elucidate the clinical features of HIT.

## Methods

### Study design

A prospective multicentre cohort study.

### Subjects and settings

This study was conducted in three Japanese stroke centres at the then National Cardiovascular Centre (currently the National Cerebral and Cardiovascular Centre, Osaka), Research Institute for Brain and Blood Vessels Akita (Akita), and Kumamoto University (Kumamoto). Between October 2006 and May 2007, all consecutive patients who met the following criteria were enrolled. Eligible patients were 20 years of age or older and admitted within 7 d after the onset of acute ischaemic stroke, including cerebral infarction and transient ischaemic attack. Patients were excluded for any of the following: (i) active infectious endocarditis, (ii) urgent neurosurgery or cardiovascular surgery would be required, (iii) chronic thrombocytopenia (defined as a platelet count <100 × 10^9^/l for more than 30 d), (iv) haematopoietic malignancy and (v) an ongoing need for an anticancer-drug treatment. The study was approved by the research ethics committee of each centre. Heparin therapy was provided to a number of patients depending on the physician's decision (mainly considering the type of stroke and/or the patient's clinical status as described in the Introduction.)

### Evaluation

The following patient characteristics were obtained: age, sex, height, body weight, body-mass index, modified Rankin Scale (mRS) score (van Swieten *et al*, 1988) before stroke onset, vascular risk factors (hypertension, diabetes mellitus, dyslipidaemia, current and past smoking habits, drinking habit, including occasional drinking), past history (autoimmune disease, haemodialysis, renal dysfunction, angina, myocardial infarction, cerebral infarction, transient ischaemic attack, pulmonary thromboembolism, extremity gangrene, amputation of an extremity, angiography, heparin exposure, surgical procedure and HIT), platelet counts, antiplatelet/anticoagulant drug use and blood transfusions. The timing and period of heparin administration (including heparin flushes), changes in platelet count, and alternative anticoagulant therapy for HIT (if given) were also examined. Other risk factors for stroke, such as emboligenic heart diseases including atrial fibrillation, were assessed based on the criteria from the Trial of Org 10172 in Acute Stroke Treatment (TOAST) study (Adams *et al*, 1993). Based on the neurological, radiological, cardiological and haematological profiles, the stroke subtype was determined according to the TOAST subtype classification system by a consensus of stroke neurologists. The neurological severity of each patient was assessed by an experienced stroke neurologist according to the National Institutes of Health Stroke Scale (NIHSS) score (Lyden *et al*, 1994) on admission and discharge, and at 3 months after onset. Patient global outcome was also assessed with mRS (van Swieten *et al*, 1988).

#### Clinical evaluation

The clinical probability of HIT was assessed using the 4Ts scoring system (Warkentin & Heddle, 2003), which is composed of four clinical features that are given scores of 0, 1, or 2; magnitude of thrombocytopenia; timing of platelet count fall (in relation to heparin therapy); thrombosis or other sequelae; and presence of other explanations for thrombocytopenia. The case reports of the patients, filled out by their physicians, were assessed independently in a blinded fashion by the external Data Assessment Committee, which consisted of two stroke neurologists, according to the 4Ts scoring system after the patient follow-up was completed. If the judgment was not concordant between the two stroke neurologists, they discussed the cases to reach a final consensus and decision. Based on the 4Ts score, the estimated pretest probabilities of HIT were categorized into three groups: low (0–3), intermediate (4–5) and high (6–8) scores. We diagnosed the patients with an intermediate or a high score as ‘potential HIT’ and those with a low score as ‘clinical non-HIT’. These objective assessments for the clinical probability of HIT were done after the patient follow-up was completed as described above, so that no results influenced clinical management. Therefore, some patients were ultimately diagnosed as HIT even though the physicians in charge did not suspect HIT as described in details in the Results section.

#### Serological evaluation

Blood samples were collected from all patients on the first (to the third) and 14th (±4) hospital days to be tested for anti-PF4/heparin Abs using ELISA (Asserachrom HPIA; Diagnostica Stago, Asnieres, France). The assays were performed in a blinded fashion after patient follow-up was completed. ELISA was performed according to the manufacturer's instructions. The titres of the samples were expressed as values of optical density (OD). The result was considered positive when the titre was greater than the cut-off value, which was determined using the reference control for each kit. To confirm the diagnosis of HIT, SRA was measured for all patients with a positive ELISA and/or ≥4 points in the 4Ts scoring system (*n* = 29). In addition, samples from 39 patients selected randomly from among all the patients were tested by SRA as a control. Samples were measured as described elsewhere at the Platelet Immunology Laboratory, McMaster University (Hamilton, ON, Canada) blinded to all clinical, platelet count and serological data (Warkentin *et al*, 1992). Any sample that produced ≥10% mean serotonin release with <10% release in the presence of high heparin (at a final concentration of 100 u/ml) and the anti-FcγRIIa monoclonal antibody (IV.3) was considered SRA-positive.

### Diagnosis

Based on the results of both the 4Ts clinical score and the serological assays, patients were categorized into four groups as follows: (i) definite HIT (4Ts score ≥4 points with positive results in both ELISA and SRA), (ii) possible HIT (4Ts score ≥4 points with positive result in either ELISA or SRA) and (iii) clinically suspected HIT (4Ts score ≥4 points with negative results in both ELISA and SRA), seropositive status (4Ts score <4 points with positive in both ELISA and SRA). The remaining patients were categorized as HIT unlikely.

### Statistical analysis

The variables between the groups of patients treated with and without heparin were compared using Fisher's exact test and the Wilcoxon test. For NIHSS, the change, NIHSS score at discharge minus that at admission, was also determined. Statistical analyses were performed using sas software version 9.1 (SAS Institute Inc, Cary, NC, USA).

## Results

### Patient characteristics

**Fig 1:**
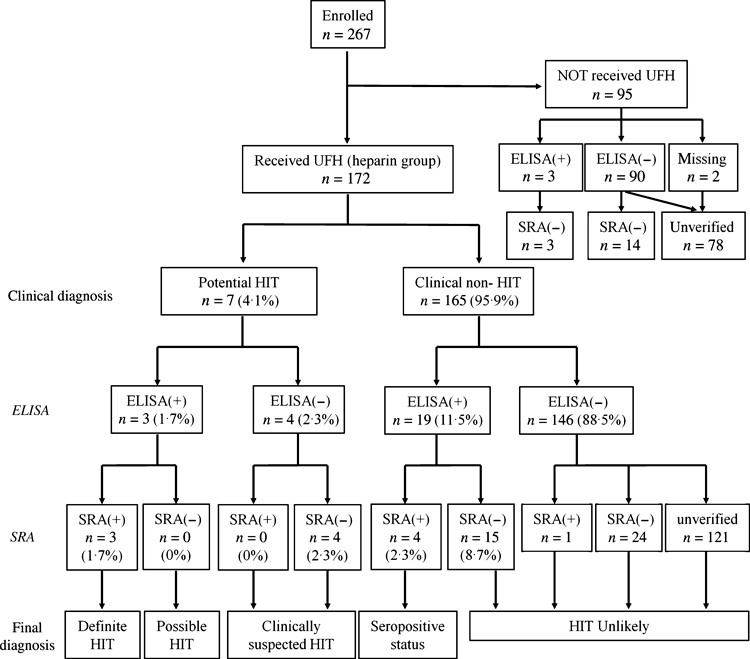
Flow chart for diagnosis of heparin-induced thrombocytopenia. HIT, heparin-induced thrombocytopenia; UFH, unfractionated heparin; ELISA, enzyme-linked immunosorbent assay; SRA, serotonin-release assay.

A total of 267 patients (mean age 71·7 years; 66·2% men), who were admitted to three stroke centres within 7 d after stroke onset during a 6-month period, were enrolled. Intravenous UFH was administered to 172 patients (64·4%: heparin group) (Fig 1). Male gender, atrial fibrillation, previous ischaemic heart disease, history of surgery using UFH, and history of intra-arterial catheter procedures were significantly more common in patients treated with than without UFH (Table IA). In regard to stroke subtype, large artery atherosclerosis and cardioembolism were more frequent in patients treated with UFH, and small vessel occlusion was more frequent in those without UFH treatment. There was no significant difference in the history of antiplatelet drug use before admission between the patients treated with (66 cases, 38·4%) and without UFH (32 cases, 33·7%) (*P* = 0·508) (Table IA). Both the NIHSS score at discharge (median, 2 vs. 1, *P* = 0·020) and mRS at 3 months after stroke onset (median, 2 vs. 1, *P*<0·001) were higher in patients treated with UFH (Table IB).

**Table I:** (A) Demographic data of patients treated or not with unfractionated heparin (UFH) and (B) clinical data of patients treated or not with UFH

	With UFH (n = 172; 64·4%)	Without UFH (n = 95; 35·6%)	*P*-value
(A)			
Age (years), median (range)	71 (23–98)	73 (42–93)	0·515
Male gender (%)	122 (70·9)	53 (55·8)	0·015
Weight (kg)	60·1 ± 12·2	59·4 ± 11·6	0·673
BMI (kg/m^2^)	23·3 ± 3·8	23·4 ± 3·7	0·936
HTN (%)	133 (77·3)	74 (77·9)	1·000
DM (%)	55 (32·0)	30 (31·6)	1·000
CRF (%)	17 (9·9)	5 (5·3)	0·247
HD (%)	3 (1·7)	0 (0)	0·555
Atrial fibrillation (%)	59 (34·3)	11 (11·6)	<0·001
Smoking (%)	78 (45·3)	37 (38·9)	0·303
Drinking (≥2 cups) (%)	49 (28·5)	21 (22·1)	0·249
Previous IHD (%)	33 (19·2)	5 (5·3)	0·002
Previous CVD (%)	51 (29·7)	28 (29·5)	1·000
Previous PTE (%)	0	0	–
Previous DVT (%)	4 (2·3)	1 (1·1)	0·658
History of heparin use within 3 months (%)	6 (3·5)	0 (0)	0·180
History of surgery using heparin	33 (19·2)	3 (3·2)	<0·001
History of intra-arterial catheter procedure (%)	43 (25·0)	8 (8·4)	<0·001
History of warfarin use (%)	18 (10·5)	5 (5·3)	0·176
History of antiplatelet agency use (%)	66 (38·4)	32 (33·7)	0·508
Stroke subtype			
TIA (%)	9 (5·2)	20 (21·1)	<0·001
Stroke (%)	163 (94·8)	75 (78·9)	
LAA (%)	38 (23·3)	5 (6·7)	
CE (%)	64 (39·3)	5 (6·7)	<0·001
SV (%)	26 (16·0)	48 (64·0)	
OT + UD (%)	35 (21·5)	17 (22·7)	
Platelet count (×10^9^/l)	222 (103–583)	230 (119–483)	0·670
NIHSS score on admission, median (range)	5 (0–32)	3 (0–20)	<0·001
(B)			
Treatment during the hospital stay			
Warfarin use (%)	70 (40·7)	9 (9·5)	<0·001
Antiplatelet agency use (%)	105 (61·0)	84 (88·4)	<0·001
Cessation of heparin (%)	142 (82·6)	0	<0·001
Alternative anticoagulation (%)	67 (39·0)	37 (38·9)	1·000
Intra-arterial catheter procedure during the hospital stay (%)	70 (40·7)	0 (0)	<0·001
Surgery with heparin use during the hospital stay	7 (4·1)	0 (0)	0·053
Thromboembolic vents or death	25 (14·5)	4 (4·2)	0·012
Recurrence of ischaemic stroke	12 (7·0)	2 (2·1)	
Thromboembolic events during catheter	4 (2·3)	0	
Other thromboembolism	7 (4·1)	2 (2·1)	
React of heparin infusion	1 (0·6)	0	
Death	5 (2·9)	0	
NIHSS score at discharge, median (range)	2 (0–42)	1 (0–20)	–
NIHSS change, discharge-admission (range)	−2 (−21 to 19)	−1 (−8 to 9)	0·020
mRS at discharge, mean (median)	2 (0–6)	1 (0–5)	0·002
mRS at 3 months, median (range)	2 (0–6)	1 (0–5)	<0·001

BMI, body mass index; HTN, hypertension; DM, diabetes mellitus; CRF, chronic renal failure; HD, haemodyalysis; IHD, ischaemic heart disease; CVD, cerebrovascular disease; PTE, pulmonary thromboembolism; DVT, deep vein thrombosis; TIA, transient ischaemic attack; LAA, large artery atherosclerosis; CE, cardioembolism; SV, small vessel occlusion; OT, stroke with alternative aetiology; UD, stroke of undetermined aetiology; UFH, unfractionated heparin; NIHSS, National Institutes of Health Stroke Scale; mRS, modified Rankin scale.

### The incidence of HIT

**Fig 2:**
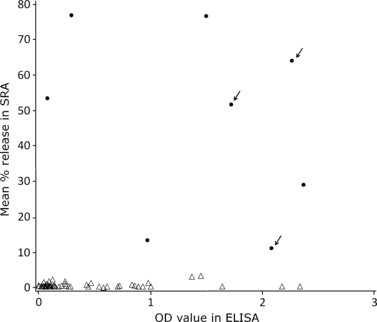
The correlation of optical density (OD) values for anti-platelet factor 4/heparin antibodies detected by enzyme-linked immunosorbent assay (ELISA) and mean percentage release by serotonin-release assay (SRA). These values showed poor correlation. Arrows indicate the data points of the three patients who met the criteria for definite HIT. •, SRA-positive cases, including one patient classed as ‘HIT unlikely': OD = 0·298, and mean percentage release = 76·74; △, SRA-negative cases.

Anti-PF4/heparin Abs were detected at any time point in 22 patients (12·8%) in the heparin group and in 3 (3·2%) of 95 patients who did not receive intravenous UFH respectively (Fig 1), and the difference was significant (*P* = 0·008). Seven patients (4·1%) were diagnosed as having potential HIT according to the 4Ts score (≥4 points). All seven patients had intermediate scores. Among them, three showed positive results in both ELISA and SRA, to give an incidence of definite HIT of 1·7% [95% confidence interval (CI): 0·4–5·0]. Possible HIT, clinically suspected HIT, and seropositive status were 0%, 2·3% (*n* = 4), and 2·3% (*n* = 4), respectively (Fig 1). Of the 95 patients with a positive ELISA who did not receive heparin within 3 months before admission and/or during hospitalization, three were SRA-negative. The OD values of anti-PF4/heparin Abs detected by ELISA seemed a little higher in definite HIT patients than the seropositive status group, although statistical analysis was not performed because of the small sample size (Table II). OD values in ELISA did not correlate with the mean percentage release in SRA (Fig 2). However, the proportion of samples with positive-SRA to those with negative-SRA was greater in the samples with ≥1·5 OD value in ELISA as compared to those with <1·5 OD value. The prevalence of positive-ELISA was not significantly different between patients who received UFH for five or more days (15·9%) and for <5 d (11·4%).

**Table II:** Clinical features of HIT patients

Pt	Age (years)	Gender	Past history	Stroke subtype	4Ts score	ELISA (OD)	SRA (mean % release)	Platelet count (×10^9^/l) Baseline	Nadir	Duration of UFH (day)	Duration of UFH up to the day of platelet nadir, days	Thrombotic complication	NIHSS on admission	mRS on discharge
Definite HIT														
1	62	Male	CI, HTN	Other	4	+(2·271)	+(63·9)	331	107	11	7	None	23	Dead
2	64	Female	CI, HTN, AF	CE	5	+(1·725)	+(51·6)	436	286	18	10	None	16	4
3	88	Female	AF	CE	5	+(2·086)	+(11·0)	156	99	7	15	None	17	Dead
Clinically suspected HIT														
4	67	Male	HTN, DM, AF, CRF	CE	4	−(0·138)	−(<1)	281	210	14	7	DVT	7	4
5	82	Male	CI, HTN, AF	CR	4	−(0·052)	−(<1)	137	27	1	4	None	10	4
6	66	Male	MI, HTN	CE	4	−(0·102)	−(<1)	583	225	13	17	None	12	1
7	69	Female	HTN, AF	CE	5	−(0·091)	−(<1)	297	120	23	6	RI	7	4
Seropositive status														
8	70	Female	HTN, AF	CE	0	+(1·666)*	+(53·2)	141	123	4	NA†	None	13	2
9	59	Female	HTN, AF, AID	CE	0	+(1·505)	+(76·8)	163	158	18	NA†	None	15	4
10	87	Male	IHD, HTN, AF	CE	0	+(0·977)	+(13·3)	200	150	13	NA†	None	8	5
11	90	Female	HTN, AF	CE	2	+(2·378)	+(28·8)	235	210	9	NA†	IHD	29	5

ELISA, enzyme-linked immunosorbent assay; SRA, serotonin-release assay; OD, optical density; CI, cerebral infarction; IHD, ischaemic heart disease; HTN, hypertension; DM, diabetes mellitus; AF, atrial fibrillation; CRF, chronic renal failure; MI, myocardial infarction within 4 weeks; AID, autoimmune disease; RI, renal infarction; DVT, deep vein thrombosis; other, stroke of other determined aetiology; CE, cardioembolism; NA, not applicable.

* ELISA was negative (OD: 0·079) in the sample drawn 7 d after admission, when SRA was positive. ELISA was positive (OD: 1·666) in the sample obtained 1 week later.

† Patient did not demonstrate thrombocytopenia.

### Clinical course and the treatment of definite HIT patients

Only one (Case 3) of three definite HIT patients was suspected of having HIT by the treating physician. This patient had atrial fibrillation and an infarct in the right anterior and middle cerebral arteries. The admission NIHSS score was 17 (Table II). The patient's platelet count decreased from 156 to 99 × 10^9^/l (approximately a 37% fall) in the typical HIT window (5–10 d) and recovered to 227 × 10^9^/l soon after stopping heparin administration on day 7 due to the suspicion of HIT. The patient had a further fall in platelet count, from 227 to 99 × 10^9^/l (approximately a 56% fall), after day 10 with a high OD value (2·086) in ELISA and a weak positive SRA (11% release) (Table II). The patient died due to deterioration from an underlying stroke. The very weak SRA, which was performed during the second platelet count fall, argues somewhat against this patient having HIT. However, HIT antibodies sometimes become weaker very quickly (Warkentin & Kelton, 2001; Greinacher *et al*, 2009), and so it is possible that the SRA would have been stronger during the first platelet count fall.

The other two patients (Cases 1 and 2) that ultimately met the criteria for definite HIT in this study were not suspected of having HIT by their physicians. One patient (Case 1) experienced a stroke of other determined aetiology due to arterial dissection in the intracranial left vertebral artery. The admission NIHSS score was 23 (Table II). The patient had bilateral cerebellar and brain stem infarcts. UFH was administrated for 7 d, and UFH flushes for intravascular catheter were continued for an additional 4 d. The patient showed a 52·0% decrease in platelet count, from 331 to 107 × 10^9^/l, that began on day 5 of heparin with relatively high values in SRA (63·9% release) and ELISA (2·271 OD value) (Table II). Death occurred from stroke on day 11. The other patient (Case 2) with a previous history of recent transient ischaemic attacks had a cardioembolic stroke due to atrial fibrillation 9 d after urgent hemiarch replacement due to aortic dissection. The admission NIHSS score was 16. The patient's platelet count declined from 436 to 286 × 10^9^/l (a drop of approximately 34%) during the typical HIT window of days 5–10 with relatively high values in SRA (51·6% release) and ELISA (1·725 OD value); although the platelet count evolution may be explained by a platelet count profile of post-cardiovascular surgery with cardiopulmonary bypass overshooting around postoperative day 14 and returning gradually to the baseline (Table II). The patient was dependent at discharge and at 3-month follow-up.

None of the patients in this study met the diagnosis of rapid or delayed onset HIT. None of the patients classified as definite HIT received treatment with alternative anticoagulants, such as thrombin inhibitors, nor did the patients develop additional thromboembolic events.

## Discussion

HIT should be recognized as a clinicopathological syndrome because none of the currently available HIT diagnostic tools have sufficient sensitivity and specificity to be used as the primary or only tool to diagnose HIT. Thus, both clinical and serological diagnoses are crucial. In this prospective study, clinical probability was assessed using the 4Ts scoring system, which is a popular method, by two independent stroke neurologists who were blinded from the results of serological assays. As a result, 4·1% of the acute stroke patients treated with heparin were suspected clinically of having HIT with ≥4 points in the 4Ts scoring system. Among them, 1·7% (95% CI: 0·4–5·0) had platelet activating antibodies against the complexes of PF4 and heparin detected by ELISA and SRA, supporting the diagnosis of definite HIT. All of these definite HIT patients had intermediate scores in the 4Ts as well as four clinically suspected HIT cases, as shown in Table II. Thus, it was very difficult to distinguish HIT patients from non-HIT patients through clinical information alone. This may possible explain why only one among three definite HIT cases was suspected of having HIT by the treating physicians.

Our results were similar to those reported in other studies of patients with ischaemic stroke (Ramirez-Lassepas *et al*, 1984; Harbrecht *et al*, 2004) and the frequency of definite HIT was less than in surgical patients (Kappers-Klunne *et al*, 1997; Warkentin, 2007b). For two of the three definite HIT patients reported here, one had a possible alternative aetiology that could explain her platelet count fall (Case 2) and the other had a weak positive-SRA (Case 1) as described in detail in the Result section. Thus, we cannot exclude the possibility that these two patients might not have had HIT. If we exclude these patients, the incidence of HIT could be as low as 0·6%. However, this result was compatible with our previous retrospective study of the same patient population (the incident of HIT was 0·5%) (Kawano *et al*, 2008). Therefore, we can conclude that the incidence of HIT in acute stroke patients treated with UFH seems to be approximately 0·5–1·7%. These results emphasize that HIT diagnosis should be considered in the management of acute ischaemic stroke.

Another major finding was that the clinical severity and outcome of acute stroke patients who were diagnosed as having definite HIT were unfavourable. In particular, the in-hospital mortality of definite HIT was very high (66·7%). Previous reports also indicated that mortality was high in HIT patients (Warkentin *et al*, 1995, 2000; Kappers-Klunne *et al*, 1997). The present study is unique in that initial neurological severity and clinical outcomes of stroke patients with HIT were determined. The NIHSS score on admission (median, 17) in definite HIT was quite high, and the outcome at 90 d was poor. However, the poor outcome of those patients appeared to be mainly due to the severity of the initial stroke rather than HIT. Although clinical severity and outcome of patients treated with UFH were unfavourable compared to those without UFH, the patients with UFH intrinsically might be at high risk of thromboembolic complications because those patients more frequently had systemic atherosclerotic changes or embolic sources. In fact, stroke subtypes were distributed differently between patients with and without UFH in our study. Hoh *et al* (2005) reported significantly less favourable outcomes, including new thromboembolic episodes and deaths in patients with subarachnoid haemorrhage who developed HIT compared to those without HIT. They found that more patients with HIT showed a poorer Fisher Grade than those without HIT, although the diagnosis of HIT was based on clinical criteria, and serological examinations were not mandatory in the study (Hoh *et al*, 2005). It should be considered that serious neurological conditions might be vulnerable to HIT.

In the present study, four of 165 clinical non-HIT patients were positive by both ELISA and SRA. None of these patients demonstrated thrombocytopenia, nor did they die. A thromboembolic event occurred in one patient who developed an ischaemic heart event. Previous reports suggested that high OD values in ELISA and/or strong-positive SRA results were associated with a high degree of diagnostic accuracy for HIT (Warkentin *et al*, 1995, 2008; Lo *et al*, 2007). However, despite high OD values (≥1·5 units) in ELISA (Cases 8, 9, 11) or strong-positive (≥50% serotonin release) SRA results (Cases 8, 9), these patients did not develop HIT (Table II). One of the clinical non-HIT patients was ELISA-negative but SRA-positive and did not develop any thrombocytopenia, thromboembolic event, or death. Furthermore, three of 95 patients without UFH were positive only by ELISA. In the present study, we blindly evaluated anti-PF4/heparin Abs in all clinical HIT and clinical non-HIT patients. Even if the results of anti-PF4/heparin Abs were positive, all patients with positive results would not always demonstrate HIT, and some of the positive results might not be pathological findings. Therefore, we should be aware of false negative and false positive results in both serological tests, and that diagnosis by the detection of anti-PF4/heparin Abs alone (even with a high OD value in ELISA and/or a strong-positive SRA result) can result in an overdiagnosis of HIT.

This study had some limitations. First, none of the patients underwent venous ultrasound; therefore, subclinical DVT, which is the typical thrombotic complication associated with HIT, may have been underdiagnosed. Second, the dose of UFH could be a determinant for the occurrence of HIT, as stoichiometrically optimal ratios of PF4:heparin influence immunization (Greinacher *et al*, 2008; Warkentin *et al*, 2010). However, in the present study, the dose and blood levels of UFH were not investigated.

In conclusion, the incidence of definite HIT in acute ischaemic stroke patients treated with UFH was 1·7% (95% CI: 0·4–5·0). HIT should be recognized as a clinicopathological syndrome in which both the clinical profile consistent with HIT and the results of serological tests should be carefully considered for HIT diagnosis. The clinical severity and outcome of acute stroke patients who were diagnosed as having definite HIT were unfavourable.
